# Transcriptome Sequencing and Differential Expression Analysis of Ovaries from Kazakh Mares During Seasonal Quiescence and Estrus Activation

**DOI:** 10.3390/biology15171566

**Published:** 2026-09-07

**Authors:** Yuhe Zhou, Wanlu Ren, Yaqi Zeng, Jianwen Wang, Jun Meng, Xinkui Yao, Manjun Zhai

**Affiliations:** 1 College and of Animal Science, Xinjiang Agricultural University, Urumqi 830052, China; 2 Xinjiang Key Laboratory of Equine Breeding and Exercise Physiology, Urumqi 830052, China

**Keywords:** Kazakh horse, ovary, transcriptome sequencing, differential expression analysis

## Abstract

Kazakh horses are typical seasonal breeders, and ovarian activity is markedly reduced during the non-breeding period. Hormonal stimulation can induce ovarian functional responses during seasonal anestrus; however, the early ovarian transcriptional changes associated with this response remain incompletely understood. In this study, ovarian transcriptome sequencing was performed in 12 Kazakh mares examined during the seasonal anestrous period. The mares were classified into a non-hormonally stimulated seasonal quiescent group (DB, *n* = 6) and a hormone-induced ovarian activation group (DY, *n* = 6) following cloprostenol and eCG treatment. RNA sequencing and bioinformatics analyses were conducted to identify differentially expressed genes and enriched biological pathways. Using false-discovery-rate-corrected criteria (FDR < 0.05 and |log2FC| > 1), a total of 2119 differentially expressed mRNAs and 530 differentially expressed lncRNAs were identified between the two groups. FDR-corrected GO and KEGG enrichment analyses revealed significant changes associated with immune regulation, cellular communication, metabolic processes, and reproductive functions. KEGG pathway analysis demonstrated significant enrichment of complement and coagulation cascades, cytokine–cytokine receptor interaction, chemokine signaling pathway, PI3K–Akt signaling pathway, and ovarian steroidogenesis-related pathways (q ≤ 0.05). *C1QB*, *CREB5*, and *IRS1* were prioritized as transcriptomic candidate genes based on their differential expression and pathway associations, but their functional roles were not experimentally tested. RT-qPCR analysis of selected DEGs showed expression trends concordant with the RNA-seq results and was used as a technical consistency assessment. Because the DY group was pharmacologically induced during seasonal anestrus and tissues were collected shortly after ultrasound confirmation of ovarian activation, the observed transcriptional profile should be interpreted as an early hormone-responsive state rather than a stable estrous state. Furthermore, because untreated naturally cycling mares during the reproductive season were not included, endogenous seasonal effects cannot be distinguished from pharmacological effects of hormonal stimulation.

## Simple Summary

Kazakh horses are typical seasonal breeders, and their reproductive activity is strongly influenced by seasonal environmental changes. During the non-breeding season, ovarian activity is markedly reduced. Hormonal treatment can induce an ovarian response during this period, but the associated early molecular changes remain incompletely understood. In this study, we compared ovarian gene expression between non-hormonally stimulated seasonally quiescent mares and mares showing ovarian activation following cloprostenol and eCG treatment during the same seasonal anestrous period. Numerous differentially expressed genes were associated with immune regulation, metabolic processes, and reproductive functions. *C1QB*, *CREB5*, and *IRS1* were highlighted as transcriptomic candidate genes based on their differential expression and association with relevant biological pathways; however, their functional roles were not tested in this study. Importantly, the hormonally induced group does not represent the natural transition to breeding-season reproductive activity or a stable natural estrous state. The observed transcriptional differences may therefore reflect both ovarian functional responses and direct or indirect effects of hormonal treatment. These findings should be considered a basis for further hypothesis-driven studies rather than evidence of specific molecular mechanisms controlling natural seasonal reproduction.

## 1. Introduction

Horses are typical seasonal breeders, and their reproductive activities are strongly influenced by environmental factors. In general, mares exhibit reproductive activity mainly during spring and summer, whereas autumn and winter are characterized by seasonal anestrus, a physiological state associated with reduced reproductive activity and suppressed ovarian function [1]. Seasonal ovarian quiescence therefore represents more than a simple reduction in follicular activity; it is a physiological state accompanied by coordinated changes in endocrine responsiveness, cellular signaling, metabolism, and local ovarian tissue interactions. Conversely, acquisition of an ovarian responsive state requires coordinated restoration of follicular growth, steroidogenic activity, intercellular communication, and tissue remodeling. Characterizing the associated transcriptional changes is therefore important for identifying the molecular processes through which a quiescent ovary becomes responsive to reproductive stimulation [2,3]. The Kazakh horse is an important indigenous breed in China, characterized by a large population, wide distribution, stable genetic performance, strong environmental adaptability, and excellent tolerance to roughage [4]. Similar to other seasonal breeding animals, reproductive activities of Kazakh horses also exhibit distinct seasonal patterns. As long-day seasonal polyestrous equines, Kazakh mares show markedly reduced ovarian activity during the winter anestrous period, whereas recurrent follicular development and cyclic ovulation resume during the natural breeding season [5]. However, natural breeding-season activity and pharmacologically induced ovarian activity during seasonal anestrus represent physiologically different conditions and should not be considered equivalent.

The ovary is a central organ involved in female reproductive regulation, responsible for follicular development, oocyte maturation, and the synthesis and secretion of steroid hormones, including estrogen and progesterone, which are essential for maintaining reproductive activity and endocrine homeostasis [6]. Ovarian function is regulated by both endogenous hormonal signals and environmental cues, resulting in dynamic changes in ovarian functional status according to reproductive stage and environmental conditions [7]. In seasonal breeding animals, ovarian activity undergoes a transition between reproductive activation during the breeding season and seasonal quiescence during the non-breeding season. This process involves coordinated regulation of follicular development, steroidogenesis, and multiple cellular signaling pathways [8].

Transcriptomic analysis provides a useful molecular framework for examining these coordinated ovarian changes because differences in gene expression can reveal biological processes and signaling pathways that distinguish quiescent from responsive ovarian states. Such molecular characterization is particularly relevant in seasonally breeding mares, for which the ovarian transcriptional responses associated with reactivation during anestrus remain incompletely understood [9], as stage-specific gene expression patterns determine ovarian functional changes and endocrine responses [10,11,12]. Previous studies have investigated the molecular mechanisms underlying reproductive state transitions in various animal species. An et al. [13] employed whole-transcriptome sequencing to characterize gene expression profiles in goat ovaries at different reproductive stages and identified significant alterations in key genes, including *MMP9*, *TIMP1*, *3β-HSD*, and *PTGIS*. These genes were involved in follicular development, steroid hormone synthesis, and luteal function maintenance, indicating that changes in ovarian functional status are accompanied by extensive transcriptional remodeling. In addition to local ovarian regulation, reproductive activity is also controlled by the hypothalamic–pituitary–gonadal (HPG) axis. Salehi et al. [14] reported that the expression levels of *KiSS1* and *RFRP-3* in the rat hypothalamus exhibited dynamic changes during the reproductive cycle. *KiSS1* promotes GnRH secretion, whereas *RFRP-3* suppresses GnRH release, and their coordinated regulation contributes to reproductive rhythm control. These findings demonstrate that reproductive state transitions are regulated by complex interactions between ovarian function and neuroendocrine signaling networks.

Although previous studies have provided valuable insights into reproductive regulation in different animal species, the early ovarian transcriptional response to experimentally induced hormonal activation during seasonal anestrus remains insufficiently characterized in horses. Therefore, the present study compared ovarian tissues from non-hormonally stimulated seasonally quiescent Kazakh mares (DB group) with those from mares showing ovarian activation following cloprostenol and eCG treatment during the same seasonal anestrous period (DY group). Through transcriptome sequencing and bioinformatics analyses, we aimed to characterize transcriptome-wide molecular differences between seasonal ovarian quiescence and an experimentally induced early ovarian responsive state, with particular attention to biological processes involving immune regulation, cellular communication, metabolism, and reproductive function. Importantly, this experimental design was not intended to reproduce the natural transition from seasonal anestrus to breeding-season reproductive activity. Because no untreated naturally cycling reproductive-season group was included, endogenous seasonal effects cannot be separated from direct or indirect pharmacological effects of hormonal stimulation. Natural breeding-season ovarian activity develops through coordinated seasonal neuroendocrine and ovarian regulation, whereas the DY condition in the present study was experimentally induced by cloprostenol and eCG during seasonal anestrus. Therefore, the ovarian morphology and estrous behavioral signs observed in the DY mares should not be interpreted as evidence that the induced condition is physiologically equivalent to naturally occurring estrus. Accordingly, the present study focuses on transcriptional changes associated with an early hormone-induced ovarian response under seasonal anestrous conditions rather than on molecular mechanisms governing natural seasonal reproductive activation.

## 2. Materials and Methods

### 2.1. Experimental Animals and Sample Collection

This experiment was conducted in Tacheng Prefecture, Xinjiang, China, in February 2025. February corresponds to the seasonal anestrous period of local Kazakh horses. To obtain mares showing a hormone-induced ovarian response (DY group), hormonal treatment was performed during this seasonal anestrous period. Each DY mare received an intramuscular injection of 0.2 mg cloprostenol, followed by an injection of 1000 IU equine chorionic gonadotropin (eCG) 7 days later. Transrectal ultrasonography was conducted daily to monitor ovarian follicular development. Mares exhibiting a dominant preovulatory follicle (≥35 mm in diameter) accompanied by typical estrous behavioral signs following hormonal treatment were classified as the hormone-induced ovarian activation group (DY group). In contrast, mares without hormonal induction that lacked dominant follicles and showed persistent ovarian quiescence were classified as the seasonal quiescent group (DB group). Once the predefined ovarian activation criteria were confirmed by ultrasonography, ovarian tissues were collected within 12 h after this ultrasound confirmation. The exact individual interval from eCG administration to tissue collection and the precise interval from the initial emergence of a dominant follicle to tissue collection were not prospectively recorded. Thus, the DY group represents an early hormone-responsive ovarian condition established during seasonal anestrus rather than a naturally occurring breeding-season or stable estrous state. A total of 12 mares with comparable reproductive conditions were selected and classified into the hormone-induced ovarian activation group (DY group, *n* = 6) and seasonal quiescent group (DB group, *n* = 6). Ovarian tissues were collected immediately after slaughter and rapidly frozen in liquid nitrogen for subsequent analysis. All horses were housed in the same facility but in separate compartments and were provided with a uniform diet consisting of high-quality dry alfalfa and corn kernels, with free access to sufficient drinking water.

### 2.2. RNA Extraction, Quality Assessment, Library Construction, and Sequencing

Total RNA was extracted from frozen ovarian tissues using a Trizol-based phenol–chloroform procedure. Briefly, ovarian tissues were ground under liquid-nitrogen conditions and lysed in Trizol reagent. Following phase separation with chloroform, the aqueous phase was collected, RNA was precipitated with isopropanol, washed with 75% ethanol, dissolved in RNase-free water, and stored at −80 °C until further analysis [15]. RNA purity and integrity were evaluated using agarose gel electrophoresis, a NanoDrop 2000 spectrophotometer (Thermo Fisher Scientific, Waltham, MA, USA), and an Agilent 2100 Bioanalyzer (Agilent Technologies, Santa Clara, CA, USA). Only RNA samples meeting the required quality criteria were used for library construction. For strand-specific transcriptome library preparation, ribosomal RNA was depleted from total RNA, and the resulting rRNA-depleted RNA was purified and fragmented. First-strand cDNA was synthesized from the fragmented RNA, followed by second-strand cDNA synthesis using a dUTP-containing reaction system to preserve strand specificity. The double-stranded cDNA was subjected to adaptor ligation, bead-based purification, PCR amplification, and library quality assessment. Qualified libraries were sequenced on the Illumina NovaSeq X Plus platform using paired-end 150 bp reads (PE150).

### 2.3. Data Quality Control

Quality control of the raw reads was performed using fastp (v0.18.0) [16] with the following filtering criteria: (1) reads containing adaptor sequences were removed; (2) reads containing >10% unknown nucleotides (N) were removed; (3) reads consisting entirely of adenine bases were removed; and (4) reads in which >50% of bases had quality scores ≤20 were removed. After filtering, high-quality clean reads were retained for downstream analyses. Following quality filtering, clean reads were aligned to the species-specific ribosomal RNA database using Bowtie2 (v2.2.8) [17], and reads mapped to rRNA were removed. The remaining paired-end reads were aligned to the *Equus caballus* reference genome using HISAT2 (v2.1.0) with the parameter “--rna-strandness RF” and otherwise default settings [18]. Transcript reconstruction was subsequently performed in a reference-guided manner using StringTie (v1.3.4) [19]. Reconstructed transcripts were compared with the reference annotation to identify known and novel transcript models, and novel transcript models generated during StringTie-based reconstruction were assigned MSTRG identifiers. For identification of novel lncRNAs, reconstructed transcripts ≥200 bp in length and containing at least two exons were retained for coding-potential assessment. Coding potential was evaluated using CPC2 (v2), CNCI (v0.9-r2), and FEELnc (v0.2) with default parameters. Transcripts consistently predicted to lack protein-coding potential were retained as candidate novel lncRNAs. According to their genomic positions relative to annotated protein-coding genes, novel lncRNAs were classified as intergenic, bidirectional, intronic, antisense, or sense-overlapping lncRNAs.

### 2.4. Expression Quantification and Inter-Sample Correlation Analysis

Gene and transcript abundances were quantified using Salmon (v1.10.3) [20]. Normalized expression values were used for expression-pattern visualization and inter-sample analyses, whereas raw read counts were retained for differential-expression analysis. Based on the gene expression data, principal component analysis (PCA) was performed using R software (version 4.3.1). A dimensionality reduction approach was employed to evaluate the inter-sample distance relationships, thereby revealing differences in ovarian transcriptional profiles between the DB and DY groups, as well as the within-group consistency. Concurrently, inter-sample correlation analysis was conducted by calculating the gene expression correlation coefficients between all sample pairs, and the results were visualized to present the pairwise correlations among samples.

### 2.5. Differential Expression Analysis

Differential-expression analyses of mRNAs and lncRNAs were performed separately using DESeq2 (v1.24) [21] based on raw read counts. Following normalization and statistical testing, the resulting *p*-values were corrected for multiple hypothesis testing to control the false discovery rate (FDR). The FDR-adjusted significance values, rather than nominal *p*-values, were used for differential-expression screening. Genes or transcripts with an absolute log2 fold change (|log2FC|) > 1 and an FDR < 0.05 were considered significantly differentially expressed.

### 2.6. Functional Enrichment Analysis of Differentially Expressed Genes

Functional enrichment analyses of differentially expressed genes (DEGs) were performed using the Gene Ontology (GO) database and Kyoto Encyclopedia of Genes and Genomes (KEGG) database [22]. GO enrichment analysis was used to identify overrepresented Biological Process, Cellular Component, and Molecular Function terms, whereas KEGG pathway enrichment analysis was used to identify overrepresented biological pathways. Enrichment *p*-values were corrected for multiple hypothesis testing, and GO terms or KEGG pathways with an FDR-adjusted q-value (Qvalue) ≤ 0.05 were considered significantly enriched.

### 2.7. RT-qPCR Validation

To assess concordance between the RNA-seq expression results and an independent expression assay, six differentially expressed mRNAs were examined using real-time quantitative PCR (RT-qPCR). This analysis was intended as a technical assessment of expression-trend consistency rather than targeted validation of the principal candidate genes highlighted in the functional analyses. Total RNA was extracted from ovarian tissue using RNA extraction reagent (Servicebio, G3013, Wuhan, China). Approximately 20 mg of tissue was added to 1 mL of RNA extraction reagent and thoroughly ground in a three-dimensional cryogenic grinder. Subsequently, 200 μL of chloroform substitute was added, mixed well, and centrifuged at 12,000 rpm for 10 min at 4 °C. The supernatant was collected, and RNA was precipitated with an equal volume of isopropanol, washed with 75% ethanol, and dissolved in nuclease-free water. RNA concentration and purity were determined using the NanoDrop 2000 spectrophotometer, with A260/A280 ratios between 1.8 and 2.0. One microgram of total RNA was reverse transcribed in a 20 μL reaction system using the SweScript All-in-One RT SuperMix for qPCR kit (Servicebio, G3330, China), with the program set to 25 °C for 5 min, 42 °C for 30 min, and 85 °C for 5 s. Amplification was carried out using 2X Universal Blue SYBR Green qPCR Master Mix (Servicebio, Q511, China) on a CFX Connect real-time fluorescence quantitative PCR system (Bio-Rad, Hercules, California, USA). The 20 μL reaction system included 7.5 μL of premix, 0.25 μM forward and reverse primers (sequences shown in Table S1), and 1 μL of diluted cDNA template. The amplification program was as follows: pre-denaturation at 95 °C for 30 s; followed by 40 cycles of 95 °C for 5 s and 60 °C for 30 s; and finally melt curve analysis from 65 °C to 95 °C, collecting fluorescence signals every 0.5 °C increase. All samples were run in triplicate. Relative gene expression levels were calculated using the 2^(−ΔΔCt)^ method [23], with GAPDH and β-actin as internal reference genes for normalization. For statistical analysis, Ct values from technical replicates were first averaged for each biological sample. The mean Ct value of GAPDH and β-actin was used as the reference value for normalization, and ΔCt was calculated for each biological sample. The six biological replicates per group were treated as the experimental units. Statistical comparisons between the DB and DY groups were performed on ΔCt values using a two-tailed Welch’s *t*-test, with *p* < 0.05 considered statistically significant.

## 3. Results and Analysis

### 3.1. RNA-Seq Data Analysis

In this study, 12 cDNA library-based ovarian transcriptomes were constructed (Table 1). A total of approximately 1.33 × 10^11^ high-quality reads were obtained, with an average of about 1.11 × 10^10^ reads per library. The GC content of the ovarian transcriptomes ranged from 50.74% to 53.14%. The proportions of bases with a quality score of Q20 and Q30 exceeded 99.10% and 96.34%, respectively. For all libraries, the percentage of reads mapped to the reference genome exceeded 94.61%.

### 3.2. Analysis of Expression Patterns of Ovarian Tissue Samples from Kazakh Horses

**Figure 1:**
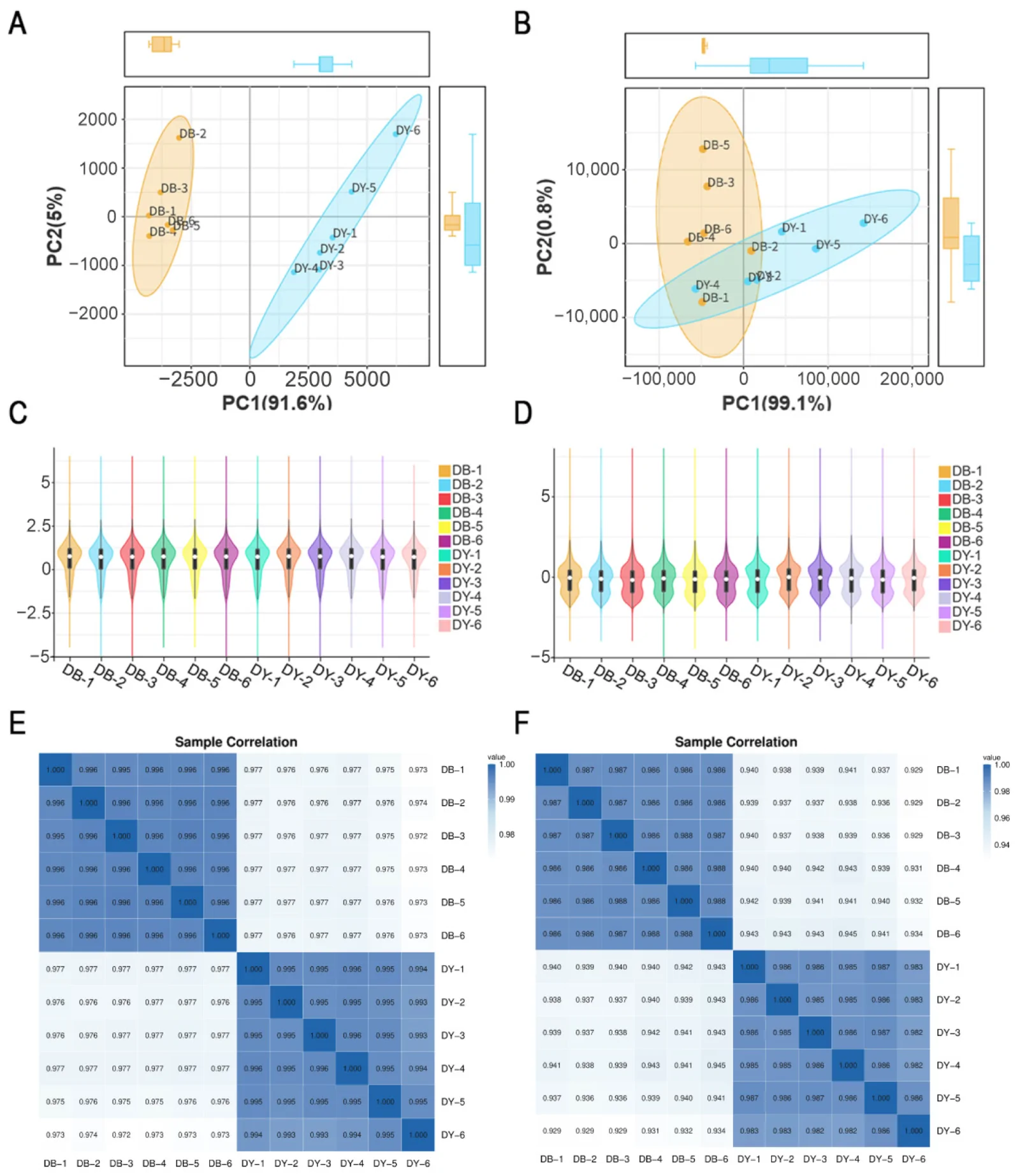
Global expression-pattern analysis of mRNAs and lncRNAs in the DB and DY ovarian samples. (**A**) Principal component analysis (PCA) of mRNA expression profiles. (**B**) PCA of lncRNA expression profiles. (**C**) Violin plots showing mRNA expression distributions across samples. (**D**) Violin plots showing lncRNA expression distributions across samples. (**E**) Inter-sample correlation heatmap based on mRNA expression profiles. (**F**) Inter-sample correlation heatmap based on lncRNA expression profiles.

In this study, we evaluated the data quality and group characteristics of the mRNA and lncRNA expression profiles using principal component analysis (PCA), expression distribution analysis, and inter-sample correlation analysis. As shown in Figure 1A,B, PCA of the mRNA expression profiles revealed that PC1 and PC2 accounted for 91.6% and 5.0% of the expression variance, respectively. Samples from the DB and DY groups were clearly separated, whereas samples within each group clustered tightly, indicating good reproducibility. In the PCA of the lncRNA expression profiles, PC1 and PC2 explained 99.1% and 0.8% of the variance, respectively, with an even more pronounced separation between the two groups. Violin plot analysis (Figure 1C,D) showed that the expression distribution patterns were consistent across all samples for both RNA types, with no systematic shifts or outlier samples. Correlation analysis (Figure 1E,F) demonstrated that the inter-sample correlation coefficients were all >0.97 for mRNAs and >0.92 for lncRNAs. Moreover, for both RNA types, the within-group correlations were higher than the between-group correlations, confirming good data reproducibility. In summary, both the mRNA and lncRNA expression profiles exhibited distinct inter-group differences, and the data quality was reliable.

### 3.3. Analysis of Differential Expression Patterns of Ovarian Tissue Samples from Kazakh Mares

**Figure 2:**
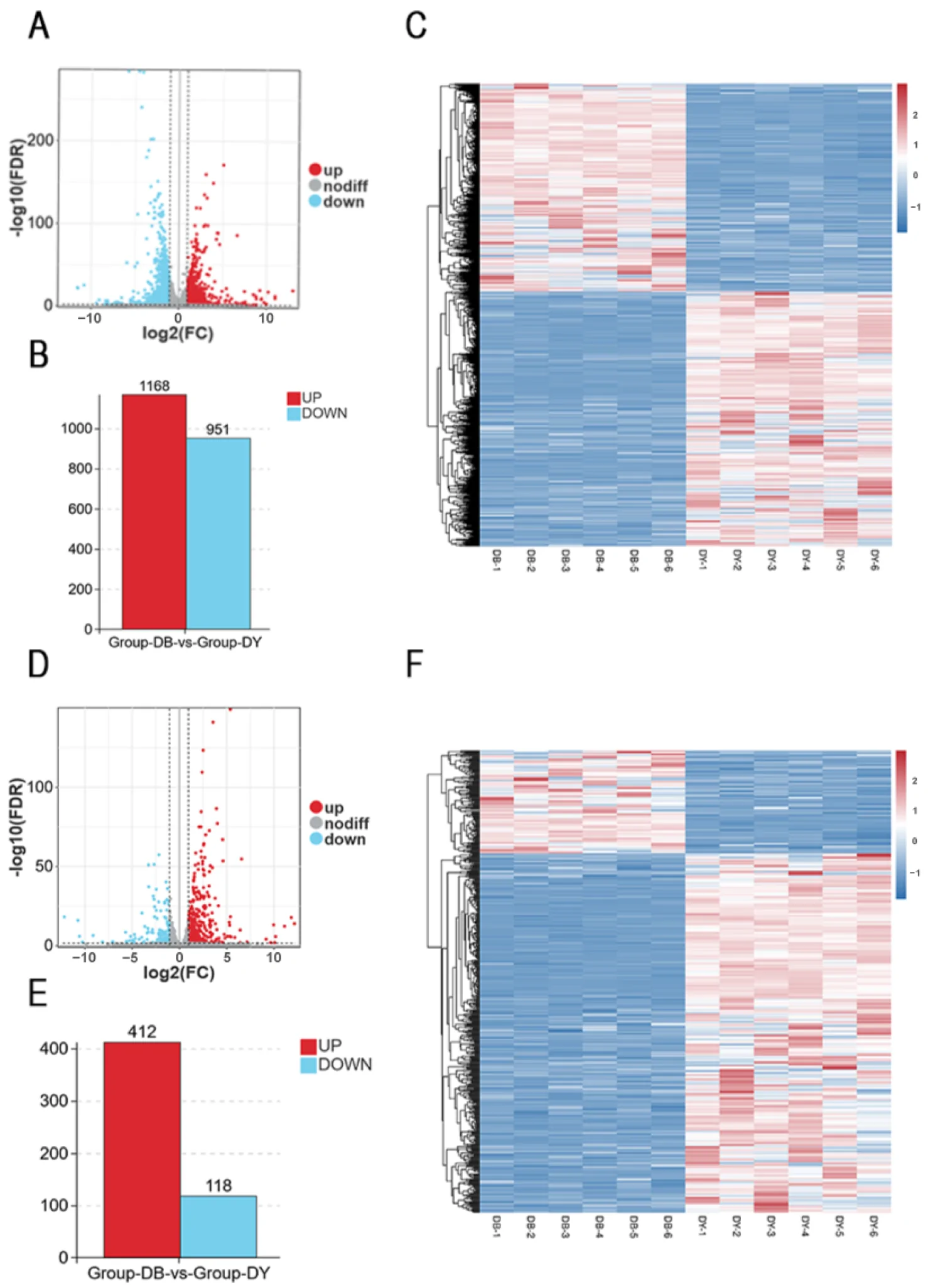
Differential-expression profiles of mRNAs and lncRNAs between the DB and DY groups. Differentially expressed transcripts were identified using FDR < 0.05 and |log2FC| > 1. (**A**) Volcano plot of differentially expressed mRNAs (DEmRNAs). (**B**) Numbers of upregulated and downregulated DEmRNAs. (**C**) Hierarchical clustering heatmap of DEmRNAs. (**D**) Volcano plot of differentially expressed lncRNAs (DELncRNAs). (**E**) Numbers of upregulated and downregulated DELncRNAs. (**F**) Hierarchical clustering heatmap of DELncRNAs.

Differential expression analysis of ovarian tissues between the two groups of Kazakh horses yielded the following results. Using FDR < 0.05 and |log2FC| > 1 as the significance criteria, a total of 2119 differentially expressed mRNAs (DEmRNAs) were identified between the DB and DY groups, of which 1168 DEmRNAs (e.g., *PLK2* and *COCH*) were upregulated and 951 DEmRNAs (e.g., *LUM* and *FOS*) were downregulated. Meanwhile, 530 differentially expressed lncRNAs (DELncRNAs) were identified (Figure 2D,E), including 412 upregulated DELncRNAs (e.g., *MSTRG.2874* and *ENSECAG00000042509*) and 118 downregulated DELncRNAs (e.g., *MSTRG.4106* and *ENSECAG00000042995*), using the same FDR-corrected significance criteria. Cluster analysis (Figure 2C,F) demonstrated distinct expression patterns between the DB and DY groups (Table S2).

### 3.4. GO Functional Annotation and KEGG Enrichment Analysis of Differentially Expressed Genes in the Ovarian Tissue of Kazakh Horses

To characterize the biological functions associated with the transcriptional differences between the two groups, GO and KEGG enrichment analyses were performed based on the DEGs identified between the DY and DB groups. Only terms or pathways meeting the FDR-adjusted significance criterion (q ≤ 0.05) were considered significantly enriched.

#### 3.4.1. GO and KEGG Enrichment Analysis of DEmRNAs in Kazakh Horse Ovaries

**Figure 3:**
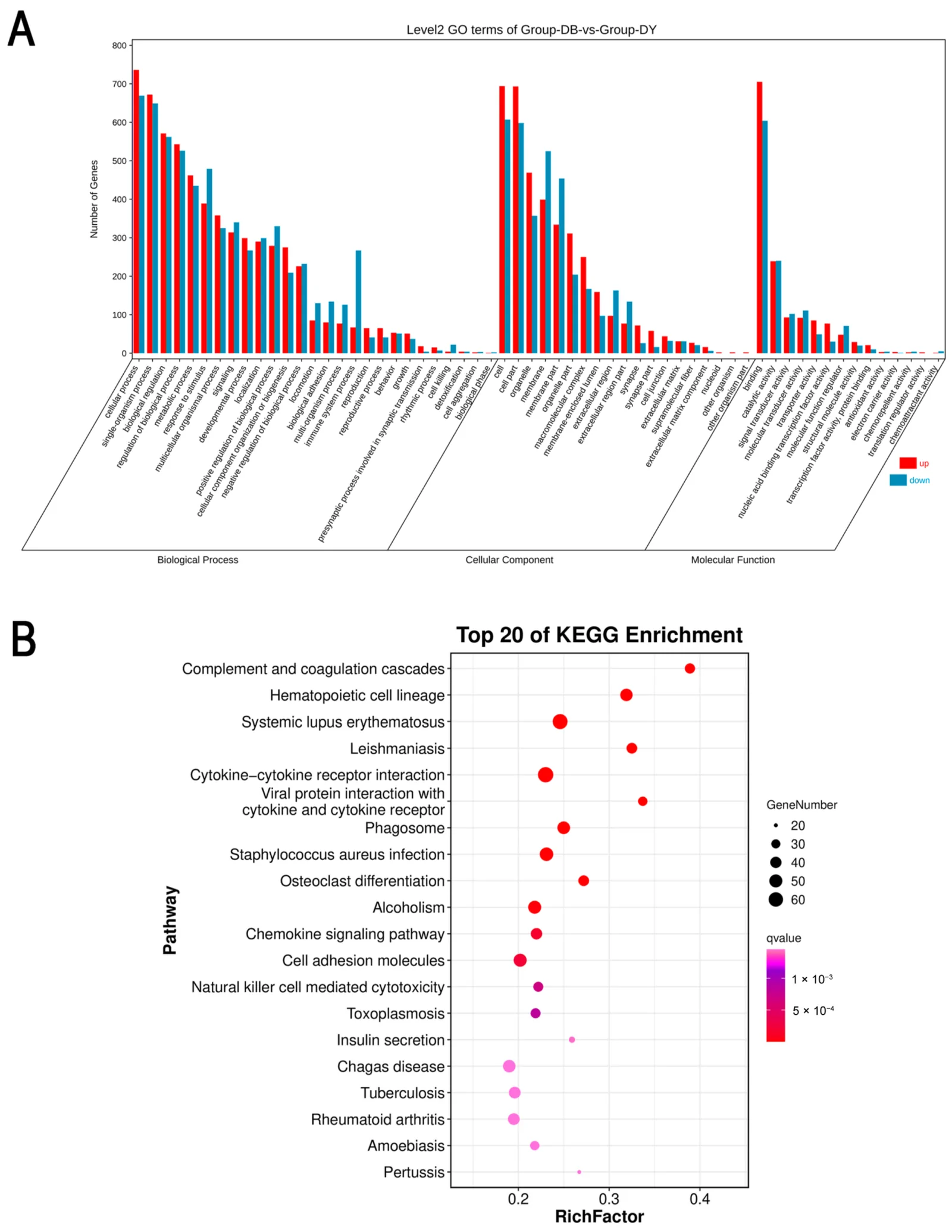
GO functional annotation and KEGG pathway enrichment of differentially expressed mRNAs between the DB and DY groups. Only terms and pathways meeting the FDR-adjusted significance criterion were considered significantly enriched. (**A**) Distribution of DEmRNAs across Gene Ontology Biological Process, Cellular Component, and Molecular Function categories. (**B**) Significantly enriched KEGG pathways of DEmRNAs.

As shown in Figure 3A, the differentially expressed mRNAs (DEmRNAs) in ovarian tissues between the DY and DB groups were significantly enriched across the three major functional categories: Biological Process, Cellular Component, and Molecular Function after correction for multiple testing (q ≤ 0.05). Within Biological Process, DEmRNAs were predominantly enriched in terms such as cellular process, single-organism process, biological regulation, metabolic process, and response to stimulus. Significant enrichment was also observed in immune system process, developmental process, and reproductive process, indicating that the transcriptional differences between non-hormonally stimulated quiescent ovaries and hormonally responsive ovaries involve genes related to immune regulation, cellular development, and reproductive function. In the Cellular Component category, DEmRNAs were mainly enriched in terms such as cell, cell part, organelle, membrane, and extracellular region. These results indicate differences in genes associated with cellular structures and extracellular interactions between the DB and DY groups, but do not establish that such changes are specific to natural seasonal ovarian activation. Within Molecular Function, DEmRNAs were primarily enriched in terms such as binding, catalytic activity, transporter activity, and signal transducer activity. KEGG enrichment analysis (Figure 3B) revealed significant enrichment of immune- and inflammation-related pathways, including complement and coagulation cascades, hematopoietic cell lineage, cytokine–cytokine receptor interaction, and chemokine signaling pathways after FDR correction. Pathways such as natural killer cell mediated cytotoxicity, phagosome, and cell adhesion molecules were also significantly enriched. The prominence of immune-related pathways represents a major transcriptional feature of the DB–DY comparison; however, because DY mares received exogenous hormonal treatment, these changes may reflect ovarian tissue remodeling, acute pharmacological responses, or a combination of both. Enrichment of pathways annotated as tuberculosis, Staphylococcus aureus infection, and toxoplasmosis likely reflects shared immune-response genes rather than evidence of pathogen exposure and should not be interpreted as a specific mechanism of ovarian activation (Table S3).

#### 3.4.2. GO Functional Annotation and KEGG Enrichment Analysis of DELncRNAs in Ovarian Tissues of Kazakh Horses

**Figure 4:**
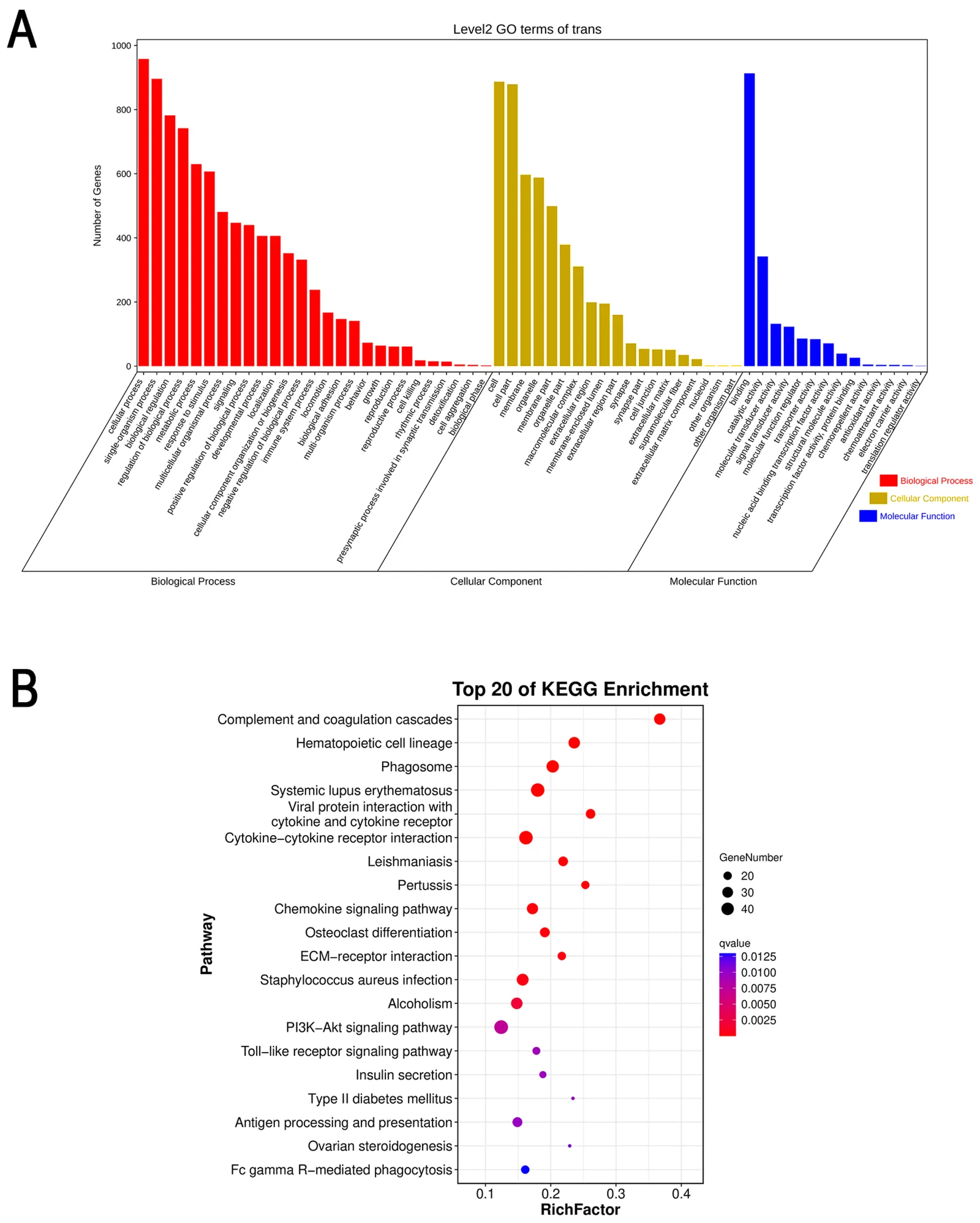
Functional enrichment of predicted target genes associated with differentially expressed lncRNAs between the DB and DY groups. (**A**) GO functional categories of predicted DELncRNA target genes. (**B**) KEGG pathways enriched among predicted DELncRNA target genes. Only enrichment results meeting the multiple-testing-corrected significance criterion were considered significant.

As shown in Figure 4A, GO analysis of the predicted target genes of differentially expressed lncRNAs (DELncRNAs) between the DB and DY groups revealed enrichment in functional terms associated with cellular processes, including cellular process (BP), single-organism process (BP), cell (CC), cell part (CC), binding (MF), and catalytic activity (MF). These results indicate that DELncRNA-associated target genes differ in functional categories related to cellular and molecular processes between the two groups. KEGG enrichment analysis (Figure 4B) showed significant enrichment of pathways including complement and coagulation cascades, cytokine–cytokine receptor interaction, chemokine signaling pathway, PI3K–Akt signaling pathway, Toll-like receptor signaling pathway, ECM–receptor interaction, and ovarian steroidogenesis after multiple-testing correction (q ≤ 0.05). These pathways are mainly associated with immune-related signaling, extracellular matrix remodeling, intracellular signal transduction, and steroid hormone synthesis. Collectively, these findings describe functional associations based on predicted DELncRNA target genes. Because lncRNA–mRNA co-expression relationships were not analyzed in the present study, these results do not establish coordinated expression or direct regulatory interactions between specific DELncRNAs and mRNAs (Table S4).

### 3.5. RT-qPCR Assessment of RNA-seq Expression Concordance

**Figure 5:**
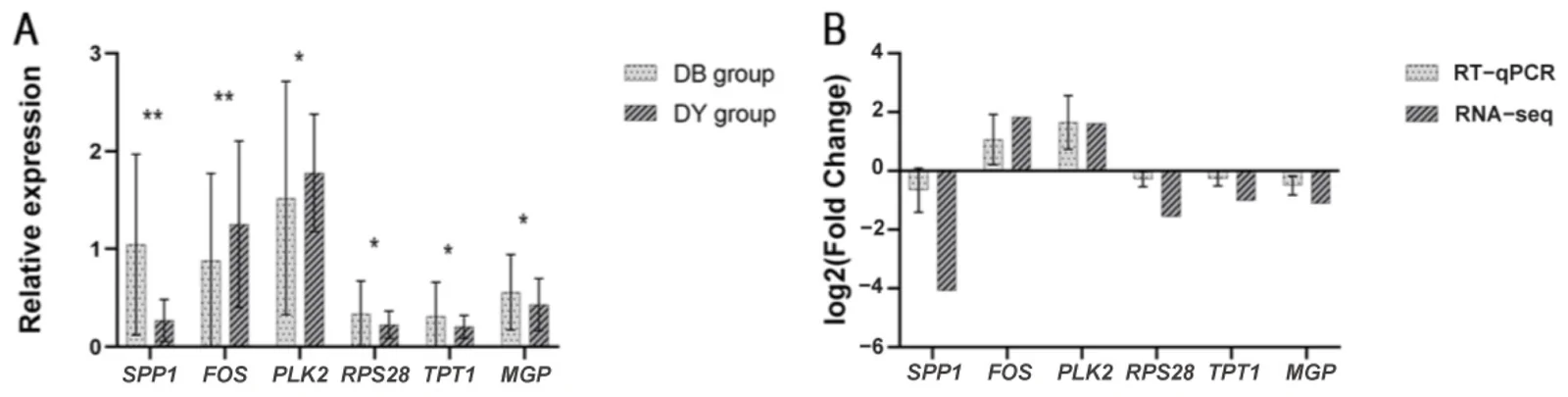
RT-qPCR assessment of RNA-seq expression concordance. (**A**) Differential gene expression detected by RT-qPCR between the DB and DY groups; (**B**) Comparison of Log2 fold change (Log2FC) of differentially expressed genes between the DB and DY groups measured by RNA-seq and RT-qPCR. Note: In the figure, “*” indicates a significant difference (*p* < 0.05), and “**” indicates a highly significant difference (*p* < 0.01).

RT-qPCR was performed for six DEGs to assess concordance with the RNA-seq results. Using *GAPDH* and *β-actin* as internal reference genes, the selected genes showed expression changes consistent with the RNA-seq dataset. Statistical analysis based on the six biological replicates per group identified significant differences between the DB and DY groups for the examined genes (Figure 5A). These results provide independent expression-level support for the RNA-seq findings.

## 4. Discussion

The principal finding of the present study is that the ovarian response to hormonal stimulation during seasonal anestrus was accompanied by broad transcriptomic remodeling rather than by isolated changes in individual genes. A total of 2119 differentially expressed mRNAs and 530 differentially expressed lncRNAs distinguished the non-hormonally stimulated seasonally quiescent ovaries from the hormonally responsive ovaries. At the functional level, these transcriptional differences converged on several interconnected biological themes, including immune regulation, cellular and extracellular communication, metabolic and growth-related signaling, and reproductive and steroidogenic processes. These transcriptome-wide patterns indicate that acquisition of an ovarian responsive state involves coordinated changes across multiple biological functions. However, because the DY condition was pharmacologically induced during seasonal anestrus, these transcriptional differences may reflect both ovarian functional remodeling and direct or indirect responses to cloprostenol/eCG treatment.

The GO enrichment results further support a broad reorganization of ovarian function. Differentially expressed mRNAs were enriched in biological processes involving cellular processes, biological regulation, metabolism, responses to stimuli, immune-system processes, development, and reproduction. Enrichment of cellular-component terms related to membranes and extracellular regions is consistent with changes in cell–cell and cell–matrix interactions, whereas enrichment of binding, catalytic, transporter, and signal-transducer functions indicates alterations in molecular processes supporting ovarian responsiveness. Rather than representing independent events, these GO categories collectively suggest changes in follicular development, metabolic support, extracellular remodeling, endocrine responsiveness, and intercellular signaling.

Among the KEGG results, immune-related pathways formed one of the most prominent functional clusters, including complement and coagulation cascades, cytokine–cytokine receptor interaction, chemokine signaling, hematopoietic cell lineage, phagosome, natural killer cell-mediated cytotoxicity, and cell-adhesion-related pathways. The ovary is an immunologically active reproductive tissue, and follicular development and ovulation are accompanied by immune-cell involvement, cytokine production, vascular changes, and local tissue remodeling [24]. The complement system may also contribute to reproductive tissue regulation through modulation of inflammatory responses, cellular communication, and tissue remodeling [25,26]. Accordingly, the coordinated enrichment of complement-, cytokine-, and chemokine-related pathways is compatible with remodeling of the ovarian immune microenvironment. Nevertheless, bulk ovarian RNA-seq cannot determine whether these transcriptional differences arose from altered gene expression within resident ovarian cells, changes in the abundance or activation state of immune-cell populations, or a combination of these processes. Moreover, because the DY mares received exogenous hormonal stimulation, part of the immune-related signature may represent an acute direct or indirect pharmacological response rather than a component specific to natural seasonal ovarian reactivation. Within this broader complement-associated transcriptional signature, *C1QB* was identified as a differentially expressed candidate. *C1QB* encodes the B chain of complement component C1q and, together with *C1QA* and *C1QC*, contributes to formation of the C1q complex that initiates the classical complement pathway [27]. C1q-associated expression has been linked to macrophage-rich cellular environments [28], while ovarian macrophages are known to participate in follicular development and tissue remodeling through the production of cytokines and growth factors [29,30,31]. These observations provide biological context for the *C1QB*-associated signal identified in the present dataset. However, the bulk-tissue RNA-seq data do not establish that the altered *C1QB* expression originated from macrophages, demonstrate a change in macrophage abundance, or show that *C1QB* directly regulates ovarian activation. The observed *C1QB* difference may therefore reflect complement activity, immune-cell composition, tissue remodeling, pharmacological stimulation, or a combination of these factors. Accordingly, *C1QB* should be regarded as one candidate within the broader complement-associated transcriptional response rather than as a demonstrated regulator of ovarian activation.

Metabolic and cellular adaptation represented another important component of the transcriptomic differences between the DB and DY groups. Follicular growth and steroidogenic activity impose substantial energetic and biosynthetic demands, and transcriptional regulators responsive to hormonal and metabolic cues may participate in coordinating these changes. *CREB*-family signaling is involved in cellular responses to intracellular and extracellular signals and contributes to the regulation of proliferation, differentiation, metabolic homeostasis, and endocrine responses [32,33,34,35,36]. Alterations in *CREB*-related expression have also been reported under conditions involving changes in metabolic and insulin-related signaling [37]. In the present study, *CREB5* was differentially expressed and was associated with cellular and metabolic signaling processes, providing the basis for its prioritization as a transcriptomic candidate. However, evidence linking *CREB*-related mechanisms with ovarian physiology is derived largely from non-equine systems, including mouse ovarian granulosa cells and human reproductive/pathological models [38]. These cross-species observations provide biological context but cannot establish an equivalent role in Kazakh mares. *CREB5* should therefore be interpreted as a transcriptomically prioritized candidate associated with altered cellular and metabolic signaling rather than as a demonstrated driver of ovarian activation.

In parallel with immune-associated and metabolic remodeling, enrichment of the PI3K–Akt signaling pathway indicates altered growth-, survival-, metabolic-, and endocrine-related signaling in the hormonally responsive ovaries. PI3K–Akt signaling integrates extracellular growth-factor and metabolic cues and participates in processes including cell proliferation, survival, metabolic homeostasis, and endocrine regulation [39,40,41,42]. In ovarian tissues, this signaling network has also been associated with granulosa-cell function, follicular development, and steroidogenic activity [40]. *IRS1* provides an important link between insulin/IGF-related signals and PI3K–Akt activation and therefore represents one component of this broader signaling pathway rather than an isolated candidate gene [39,40]. Experimental manipulation of *IRS1* in mouse ovarian models has been associated with changes in granulosa-cell proliferation and steroidogenic markers [43], while Akt-related signaling has also been reported to vary with reproductive and hormonal states in mice [44]. In the present study, *IRS1* expression was altered in the DY group, and its differential expression together with PI3K–Akt pathway enrichment is consistent with altered metabolic and growth-related signaling following hormonal stimulation. However, these associations do not establish a causal role for *IRS1* or PI3K–Akt signaling in Kazakh mare ovarian activation, and whether similar changes occur during naturally established breeding-season activity remains unknown.

The enrichment of ovarian steroidogenesis and reproduction-related processes provides an additional functional dimension to the immune and metabolic transcriptional signatures. Acquisition of an ovarian responsive state requires not only follicular growth but also coordinated steroidogenic activity, gonadotropin responsiveness, cellular proliferation, extracellular remodeling, and communication among the different cellular compartments of the ovary [45,46]. Therefore, the simultaneous enrichment of ovarian steroidogenesis, PI3K–Akt signaling, extracellular-matrix-related processes, and immune-associated pathways suggests coordinated endocrine, metabolic, and structural remodeling rather than a change in any single biological process. Because ovarian steroid hormone concentrations and pathway activities were not directly measured in the present study, these pathway-level findings should be interpreted as transcriptional associations rather than evidence of pathway activation.

The broad transcriptional remodeling observed in the present study also shows similarities to reproductive transcriptomic findings in other mammals. For example, whole-transcriptome analysis of goat ovaries at different reproductive stages identified extensive changes in coding and non-coding RNAs associated with follicular development, steroid hormone synthesis, and reproductive regulation [13]. In addition, experimental studies in mouse ovarian systems have implicated *CREB*-related signaling and *IRS1*-associated pathways in granulosa-cell and ovarian functions [38,44], although these findings cannot be directly extrapolated to horses [47]. Together, these cross-species observations suggest that transcriptional changes involving follicular development, metabolic signaling, and steroidogenic function may represent partly conserved components of ovarian functional remodeling. In contrast, the prominent complement-, cytokine-, and chemokine-associated signature observed in the present study may reflect a combination of ovarian biology, species or breed characteristics, tissue composition, and the specific use of pharmacological induction during seasonal anestrus [2]. Therefore, the present data do not establish that the immune-related features identified here are specific to Kazakh mares.

Taken together, the transcriptomic findings support a working model in which the early ovarian response to hormonal stimulation during seasonal anestrus involves several interconnected layers of molecular remodeling. First, changes in complement-, cytokine-, and chemokine-related pathways are consistent with alteration of the local immune and inflammatory signaling environment [48,49]. Second, changes involving extracellular and cellular communication may accompany structural remodeling and follicular development. Third, PI3K–Akt and other metabolic signaling processes may support changes in cellular growth, survival, nutrient sensing, and endocrine responsiveness [50], with *IRS1* and *CREB5* representing candidate components of these broader signaling domains. Fourth, enrichment of ovarian steroidogenesis and reproduction-related processes is consistent with concurrent restoration of follicular and endocrine functions. Within this framework, *C1QB* represents a candidate associated with the complement-related component of the transcriptional response. This integrated model is hypothesis generating and does not establish causal relationships among these pathways or candidate genes.

Several limitations should be considered when interpreting the present results. First, the biological sample size was modest (*n* = 6 per group), and both groups were examined during the same seasonal anestrous period, whereas the DY mares received cloprostenol and eCG. Because untreated naturally cycling mares during the reproductive season were not included, endogenous seasonal effects cannot be distinguished from direct or indirect pharmacological responses. Therefore, the DY group should be interpreted as an early hormone-responsive condition rather than as a natural breeding-season or stable estrous state. The exact individual interval from eCG administration to tissue collection was not prospectively recorded, which also limits assessment of inter-individual temporal variation in the ovarian response. Second, transcriptomic profiling was performed using bulk ovarian tissue; consequently, the observed expression differences cannot be assigned to specific ovarian or immune-cell populations. Third, although RT-qPCR provided statistically significant support for the expression differences of the selected DEGs, *C1QB*, *CREB5*, and *IRS1* were not included in the RT-qPCR assay and were not functionally validated. Finally, the lncRNA analysis was limited to differential-expression analysis, target-gene prediction, and functional enrichment, without lncRNA–mRNA co-expression or functional validation. Future studies incorporating larger sample sizes, appropriate seasonal controls, cell-type-resolved analyses, longitudinal sampling, and targeted functional experiments will be required to distinguish endogenous seasonal regulation from pharmacological responses and to establish the biological roles of the identified candidate genes and pathways.

## 5. Conclusions

This study characterized transcriptomic differences between non-hormonally stimulated seasonally quiescent ovaries and ovaries showing an early response to hormonal induction in Kazakh mares during the same seasonal anestrous period. FDR-corrected differential-expression and enrichment analyses identified changes involving immune-related pathways, metabolic signaling, cellular communication, and reproductive processes. *C1QB*, *CREB5*, and *IRS1* were prioritized as transcriptomic candidate genes based on their differential expression and pathway associations; however, their functional roles were not established in the present study. The prominent immune-related transcriptional signature may reflect ovarian tissue remodeling, acute responses to hormonal stimulation, or a combination of both. Because naturally cycling reproductive-season mares were not included and the DY samples represent an early hormone-responsive condition, the observed molecular differences cannot be interpreted as specific mechanisms governing natural seasonal reproductive activation or as characteristics of a stable estrous state. Further studies using appropriate seasonal controls, longitudinal sampling, targeted expression validation, and functional experiments are required to distinguish endogenous seasonal regulation from pharmacological responses and to determine the biological roles of the identified candidate genes.

## Figures and Tables

**Table 1:** Summary of RNA-seq sequencing quality and reference-genome mapping statistics for the 12 Kazakh mare ovarian samples.

Sample	Raw Data (bp)	Clean Data (bp)	Q20 (%)	Q30 (%)	GC (%)	Total_Mapped (%)
DB-1	9,616,618,500	95,096,78,251	99.22%	96.80%	52.65%	96.67%
DB-2	11,834,763,000	11,710,308,868	99.22%	96.78%	53.14%	96.71%
DB-3	11,564,623,800	11,449,439,441	99.27%	96.92%	52.32%	96.82%
DB-4	10,689,001,200	10,569,143,115	99.22%	96.74%	52.19%	96.69%
DB-5	11,359,004,100	11,214,246,520	99.21%	96.71%	52.38%	96.67%
DB-6	11,089,001,700	10,965,309,077	99.16%	96.55%	52.67%	96.60%
DY-1	12,870,671,400	12,720,101,968	99.19%	96.73%	51.41%	94.61%
DY-2	9,810,036,000	9,699,711,189	99.19%	96.70%	51.09%	95.35%
DY-3	10,153,745,700	10,043,248,224	99.17%	96.43%	50.84%	95.38%
DY-4	12,417,413,700	12,271,409,990	99.13%	96.53%	50.74%	95.32%
DY-5	13,051,215,900	12,870,150,029	99.11%	96.37%	52.06%	95.28%
DY-6	10,321,940,700	10,190,313,539	99.10%	96.34%	52.55%	95.14%

Note: Sample, sample identifier; Raw Data (bp), total number of bases in raw sequencing reads; Clean Data (bp), total number of bases retained after quality filtering; Q20 (%), percentage of bases with Phred quality scores ≥ 20; Q30 (%), percentage of bases with Phred quality scores ≥ 30; GC (%), percentage of guanine and cytosine bases; Total Mapped (%), percentage of clean reads aligned to the reference genome.

## Data Availability

The data presented in this study are openly available in BioProject with reference number PRJNA1478262.

## Supplementary Materials

The following supporting information can be downloaded at: https://www.mdpi.com/article/10.3390/biology15171566/s1, Table S1: Primers used in this study; Table S2: All differentially expressed genes; Table S3: GO and KEGG enrichment information of differentially expressed mRNA; Table S4: GO and KEGG enrichment information of differentially expressed LncRNA.
